# The Pathophysiology of Wharton’s Jelly and Its Impact on Fetal and Neonatal Outcomes: A Comprehensive Literature Review

**DOI:** 10.3390/medsci13040215

**Published:** 2025-10-02

**Authors:** Tudor-Andrei Butureanu

**Affiliations:** 1 Department of Mother and Child, Grigore T. Popa University of Medicine and Pharmacy, 700115 Iasi, Romania; tudorandreib@gmail.com; 2 “Elena Doamna” University Hospital of Obstetrics and Gynecology, 700115 Iasi, Romania

**Keywords:** wharton’s jelly, neonatal, fetal outcomes, umbilical cord

## Abstract

Wharton’s jelly (WJ), the mucoid connective tissue of the umbilical cord, provides essential protection to the umbilical vessels against mechanical stress. While research into WJ-derived stem cells for regenerative medicine has surged, the clinical significance of its in utero pathologies remains less explored. This review synthesizes the current literature on the pathophysiology of WJ abnormalities and their direct impact on fetal and neonatal outcomes. Pathologies are broadly categorized as quantitative (absence/reduction or excess/edema) and structural (pseudocysts, mucoid degeneration). A reduction or segmental absence of WJ critically compromises cord integrity, leading to vascular compression and is a direct cause of stillbirth, fetal growth restriction (FGR), and intrapartum distress. Conversely, excessive WJ or edema is associated with maternal diabetes and fetal hydrops and can also impair hemodynamics. Umbilical cord pseudocysts, arising from focal WJ degeneration, are significant markers for severe chromosomal abnormalities, particularly Trisomy 18 and 13, and other structural defects, especially when persistent or multiple. Sonographic measurement of WJ area shows promise as a surrogate for placental function, with decreased area correlating with placental pathology and FGR. However, significant diagnostic challenges persist, particularly the prenatal detection of segmental WJ absence, a “silent” pathology often discovered only after a catastrophic event. This review highlights the critical role of WJ integrity in determining perinatal outcomes and underscores the urgent need for improved diagnostic modalities and standardized management protocols to mitigate associated risks.

## 1. Introduction

Wharton’s Jelly formally known as *substantia gelatinea funiculi umbilicalis*, is a vital gelatinous substance integral to the umbilical cord’s structure and function. It was first described by the English anatomist Thomas Wharton in 1656.

A normal umbilical cord consists of two arteries and one vein, all embedded within a gelatinous, homogeneous substance known as Wharton’s jelly. The cord is covered by amniotic membranes and typically has a diameter of 1–2 cm at term. Variations in cord diameter are mostly attributed to differences in the amount of Wharton’s jelly, though the size of the blood vessels may also play a role. At full term, the cord usually reaches an average length of 55–61 cm, which is sufficient to permit vaginal delivery even when the placenta is implanted at the fundus. The umbilical cord develops throughout gestation, though its rate of growth slows after 28 weeks. Around 6 weeks post-conception, it measures about 0.5 cm in length. By the fourth month, it reaches approximately 16–18 cm, and by the sixth month, it measures about 33–35 cm.

The umbilical arteries originate from the fetal internal iliac arteries, travel alongside the fetal bladder, and exit through the umbilicus to become part of the umbilical cord. These paired arteries wrap around the umbilical vein in a helicoidal pattern, completing around 10–11 coils from the fetal to the placental end, and eventually branch out along the chorionic plate of the placenta.

The umbilical vein forms from the convergence of the chorionic veins of the placenta. Its main function is to deliver oxygenated blood to the fetus. It enters the fetus through the umbilicus and joins the left portal vein in the liver. Additionally, the umbilical vein connects to the inferior vena cava via the ductus venosus, allowing about one-third of the oxygenated blood to bypass the liver and flow directly toward the heart. After birth, the intra-abdominal segments of the umbilical vessels degenerate: the umbilical arteries become the lateral ligaments of the bladder, while the umbilical vein becomes the round ligament of the liver.

The umbilical cord is typically twisted or coiled to the left (counterclockwise), with a left-to-right coiling ratio of approximately 7:1. On average, there are about 40 coils along the entire cord, equating to roughly 0.2 coils per centimeter—a measurement known as the coiling index. Coiling begins early in gestation and can be observed via sonography as early as the 9th week. Although the exact cause of this coiling is still debated, some evidence suggests it may be influenced by fetal movements. The umbilical cord forms during the first five weeks of gestation (corresponding to seven weeks from the last menstrual period) through the fusion of the omphalomesenteric (yolk) stalk and allantoic ducts. An outgrowth from the fetal bladder creates the urachus, which extends into the connecting stalk to form the allantois. The allantoic vessels eventually develop into the definitive umbilical vessels. The cord becomes lined with epithelial cells once the amniotic cavity expands and envelops the cord with the amniotic membrane [1].

**Figure 1:**
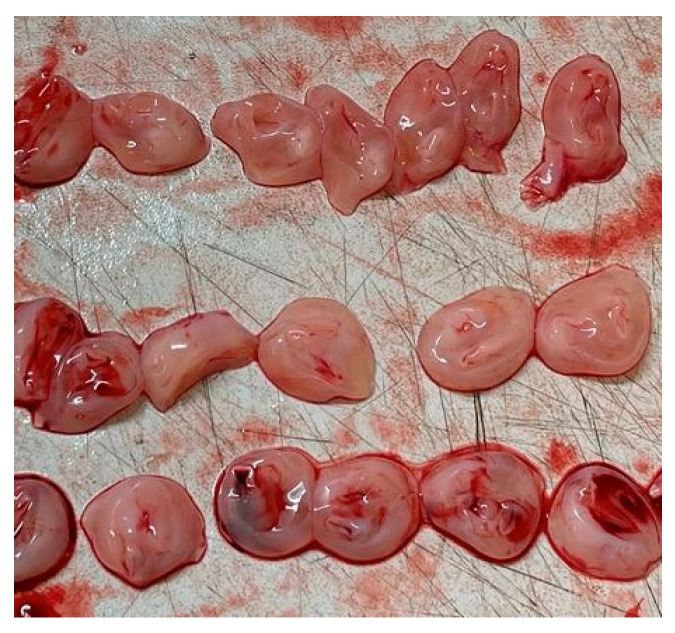
Sections of umbilical cord from a 31-week gestation fetal death showing a single artery and vein.

The primary function of the umbilical cord (UC) is to facilitate the transport of oxygenated blood and nutrients from the maternal circulation to the fetus and to return deoxygenated blood and metabolic waste products, a role indispensable for intrauterine growth, development, and survival [2]. The structural and functional integrity of the umbilical cord is therefore of great importance. A growing body of evidence demonstrates that a wide spectrum of umbilical cord abnormalities—ranging from variations in length and coiling to abnormal placental insertion and vascular anomalies—are significant contributors to adverse perinatal outcomes, including fetal growth restriction (FGR), intrapartum distress, neonatal morbidity, and stillbirth [3]. Indeed, analyses of stillbirth cohorts have attributed up to 28% of cases beyond 32 weeks’ gestation to umbilical cord anomalies, underscoring their profound clinical significance (Figure 1) [4].

**Figure 2:**
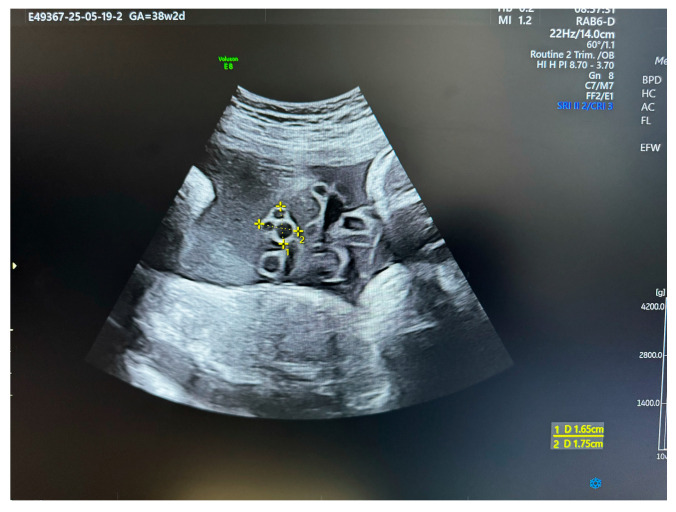
US section image of a normal umbilical cord at 38 weeks of gestation.

Central to the cord’s structural resilience is Wharton’s jelly (WJ), the specialized mucoid connective tissue that encases the umbilical vessels—typically two arteries and one vein (Figure 2) [2].

Originating from the extraembryonic mesoderm during early development, WJ constitutes the bulk of the umbilical cord and is histologically organized into distinct zones, including the subamnion, an intermediate layer, and a dense perivascular region [5]. The unique biomechanical and physiological properties of this matrix are fundamental to its protective function. Wharton’s jelly serves as a sophisticated biological cushion, safeguarding the umbilical vessels against the constant threats of compression, torsion, and bending that arise from vigorous fetal movements within the amniotic sac [2]. Its physical characteristics have been likened to polyurethane foam, providing robust resistance to external pressures and ensuring the patency of the vital circulatory lifeline [6].

This protective capacity is rooted in the complex composition of its extracellular matrix. Wharton’s jelly is rich in proteoglycans, glycosaminoglycans—with hyaluronic acid being the predominant component—and a network of collagen fibrils (Types I, II, and V) [2]. The exceptionally high concentration of hydrophilic hyaluronic acid allows the matrix to absorb and retain large amounts of water, creating a turgid, hydrated gel that maintains the cord’s architectural integrity and acts as a physical buffer for fetoplacental blood flow [7,8]. This matrix is populated by myofibroblast-like stromal cells that possess both fibrogenic and contractile properties. These cells are not merely passive structural elements; it is believed they actively participate in the regulation of umbilical blood flow through their contractile capabilities [2].

In recent decades, Wharton’s jelly has garnered immense scientific interest, but this attention has been largely bifurcated. On one hand, a vast and rapidly expanding field of research is dedicated to exploring the therapeutic potential of Wharton’s jelly-derived Mesenchymal Stromal Cells (WJ-MSCs) in regenerative medicine [9,10]. These cells are prized for their primitive nature, high proliferative capacity, and immunomodulatory properties, making them an ethically sourced and promising tool for treating a wide array of adult and pediatric diseases, from cardiovascular and neurological disorders to radiation injuries and autoimmune conditions [11]. This has led to a proliferation of preclinical studies and clinical trials, generating a substantial body of literature [10,12].

On the other hand, the literature concerning the in utero structural and pathological role of Wharton’s jelly—the very context of its biological existence—is comparatively sparse and consists primarily of case reports, small case series, and observational studies [7]. This disparity in research focus has created a significant knowledge gap. While the scientific community has made great strides in understanding how to harness WJ for therapeutic purposes ex vivo, our understanding of the fundamental mechanisms of its developmental failure in vivo and the full spectrum of its pathological implications for the fetus remains limited. Pathologies of Wharton’s jelly, though often rare, can lead to catastrophic outcomes, and their investigation is critical for advancing our understanding of pregnancy risk and the etiology of stillbirths, often vaguely attributed to “cord accidents” [2,13,14]. This review aims to bridge this gap by providing a comprehensive and systematic analysis of the existing literature on the pathology of Wharton’s jelly, focusing specifically on its direct impact on fetal and neonatal outcomes.

## 2. Methods

Searches were conducted in PubMed/MEDLINE, SCOPUS/EMBASE, and Google Scholar from 1960 to the present. Additionally, reference lists of included studies and relevant gray literature were also reviewed. Eligible studies included cohort, cross-sectional, or case–control designs involving singleton pregnancies that reported on the frequency of umbilical cord Wharton’s jelly characteristics or abnormalities and their association with stillbirth or other adverse outcomes.

### 2.1. The Spectrum of Wharton’s Jelly Pathologies

The pathologies affecting Wharton’s jelly can be broadly categorized into two main groups: quantitative abnormalities, which involve an abnormal volume or amount of the jelly, and structural or degenerative abnormalities, which involve focal lesions or changes in the matrix composition. These pathologies are not always discrete entities and may represent a continuum of developmental or degenerative processes that ultimately compromise the protective function of the umbilical cord.

### 2.2. Pathophysiology

#### 2.2.1. Reduction and Absence of Wharton’s Jelly (“Thin Cord Syndrome”)

##### Definition and Pathophysiology

A quantitative deficiency of Wharton’s jelly manifests as a “thin” umbilical cord. This condition is clinically defined by sonographic or postnatal measurements, such as an umbilical cord diameter below the 10th percentile for gestational age or a thickness of less than 0.8 cm when measured on a histological slide [15]. The underlying pathology is a significant decrease in the cross-sectional area of WJ, which is the primary determinant of a thin cord diameter [7]. This reduction in the protective matrix leaves the umbilical vessels dangerously exposed and vulnerable to external mechanical forces. Without the cushioning effect of an adequate amount of jelly, the vessels are more prone to compression from fetal parts, uterine contractions, or entanglement, which can lead to intermittent or sustained occlusion, thereby compromising fetoplacental circulation [7]. Pathological studies have identified obstructive umbilical cord lesions resulting from a decreased presence of WJ as a predominant risk factor for the development of fetal vascular malperfusion lesions within the placenta, a finding indicative of chronic circulatory compromise [2,16].

##### Etiology and Pathogenesis

The precise etiology of Wharton’s jelly deficiency is not well understood, and it is likely multifactorial. Several pathogenic mechanisms have been proposed, though they remain largely hypothetical. One theory suggests a primary developmental failure, wherein an insufficient amount of embryonal mesenchyme is allocated to the formation of the umbilical cord during early gestation [7]. A second hypothesis posits a secondary degenerative process, where normally formed WJ undergoes atrophy or breakdown later in pregnancy [7]. Other proposed mechanisms relate to defects in the formation of the cord’s outer layer, such as an incomplete fusion of the amniotic covering with the underlying umbilical mesenchyme, or hypoplasia of the amniotic covering, which could lead to a secondary loss of the gelatinous matrix [7].

##### Complete and Segmental Absence

The most extreme and life-threatening manifestation of this pathology is the complete or segmental absence of Wharton’s jelly. This is a very rare condition, with its true incidence unknown but documented through a small number of case reports in the medical literature [17,18]. While total absence is conceivable, the condition more commonly presents as a segmental defect, where a specific length of the cord, often near the fetal insertion site, is completely devoid of the protective jelly [7]. This anomaly is frequently associated with, and likely the cause of, umbilical cord constriction or stricture, a focal narrowing of the cord that can lead to complete vascular obstruction [7].

##### Association with Other Conditions

A clinically significant association has been established between a reduction in Wharton’s jelly and the presence of a single umbilical artery (SUA). In a study evaluating 22 non-malformed fetuses with a prenatally diagnosed SUA, sonographic measurements revealed that the amount of Wharton’s jelly was below two standard deviations from the mean in all 22 cases [19]. This consistent finding suggests a common underlying developmental pathology affecting the formation of both the umbilical vasculature and its supporting stroma. Additionally, alterations in WJ growth and composition have been noted in pregnancies complicated by maternal conditions such as gestational diabetes mellitus and early-onset preeclampsia, suggesting that the maternal metabolic and vascular environment can influence the development of the umbilical cord matrix [2,20].

### 2.3. Abnormalities of Pathophysiology

#### 2.3.1. Excessive Wharton’s Jelly and Umbilical Cord Edema (“Thick Cord Syndrome”)

##### Definition and Clinical Context

At the opposite end of the quantitative spectrum is an excessive accumulation of Wharton’s jelly, resulting in a “thick” or edematous umbilical cord. This is typically defined as a cord diameter greater than the 90th percentile for gestational age or exceeding 2 cm on average [15]. In extreme cases, the term “giant” umbilical cord is used, referring to a diameter greater than 5 cm [21]. While the appearance of a giant umbilical cord can be alarming, the condition is often clinically benign and harmless [22]. However, it can also be a marker for underlying maternal or fetal aneuploidies [21].

##### Histopathology and Hemodynamics

Histopathological examination of an edematous cord reveals a less dense Wharton’s jelly matrix, characterized by the swelling and separation of connective tissue fibers. In severe cases, focal hemorrhages and the formation of small and large cavities within the jelly can be observed [23]. These structural changes have significant hemodynamic consequences. A prospective study involving 90 pregnancies with umbilical cord pathology demonstrated that cases with Wharton’s jelly edema exhibited the most severe adverse alterations in the functional indices of the umbilical vessels [23]. Specifically, these cords showed a markedly increased Wagenworth index, which reflects a decrease in vessel permeability, and a significantly decreased Kernohan index, a morphological indicator of blood flow disturbance [23]. Umbilical cord edema, defined as visible swelling in a cord with a minimum cross-sectional area of 1–3 cm^2^, is observed in approximately 10% of deliveries. It occurs more frequently in association with certain pregnancy complications, such as placental abruption, maternal diabetes, and macerated intrauterine fetal death. Additionally, it is linked to several neonatal conditions, including prematurity, Rhesus isoimmunization, respiratory distress syndrome (RDS), and transient respiratory distress (TRD) [24].

These findings suggest that excessive, edematous WJ, far from being inert, can actively compress the umbilical vessels, impairing blood flow and potentially compromising fetal oxygenation.

##### Associated Conditions

Thick umbilical cords and cord edema are classically associated with several specific clinical conditions. Maternal diabetes mellitus is a well-known cause, likely related to fetal hyperglycemia and hyperinsulinemia affecting matrix production [15]. Fetal conditions such as Beckwith-Wiedemann syndrome and hydrops fetalis of any etiology are also strongly associated with an increase in Wharton’s jelly [15]. While less common, an association with chromosomal abnormalities has also been reported [22]. Furthermore, in cases of significant WJ edema, the total placental weight often deviates substantially from normative values, suggesting that the placenta’s compensatory and adaptive mechanisms may be overwhelmed or ineffective in this setting [23].

### 2.4. Structural Lesions and Degenerative Changes

#### 2.4.1. Umbilical Cord Cysts (Pseudocysts)

##### Definition and Pathogenesis

Umbilical cord cysts are among the more frequently encountered structural abnormalities of the cord. They are classified into true cysts and pseudocysts. Pseudocysts are far more common and are of direct relevance to Wharton’s jelly pathology, as they arise from a focal, localized degeneration or liquefaction of the jelly itself [25]. Histologically, they are distinguished from true cysts (which are rare embryological remnants of the allantois or omphalomesenteric duct) by their lack of an epithelial lining [26,27]. Pseudocysts can present as single or multiple lesions, can be located anywhere along the length of the cord, and vary widely in size, from a few millimeters to several centimeters in diameter [25,26].

##### Prevalence and Prognostic Significance

The overall prevalence of umbilical cord cysts is estimated to be between 0.4% and 3.4% of pregnancies, with a higher detection rate of approximately 3% during first-trimester ultrasound screening [25]. The clinical significance and prognosis of these cysts are highly dependent on several factors, including the gestational age at detection, the number of cysts, and the presence of other associated findings.

- Isolated, First-Trimester Cysts: When a single cyst is identified in the first trimester without any other sonographic abnormalities, the prognosis is generally excellent. These cysts are often transient, resolving spontaneously as the pregnancy progresses, and are typically considered a normal variant of development with a favorable outcome [25].
- Persistent, Second/Third-Trimester, and/or Multiple Cysts: The clinical implications become far more serious when cysts persist beyond the first trimester, are detected for the first time in the second or third trimester, or are multiple in number. In these scenarios, there is a strong association with underlying fetal pathology, with up to 50% of such cases having associated structural or chromosomal abnormalities [25]. The risk is further elevated if the cysts are located near the fetal or placental insertion sites of the cord [25]. These cysts are a prominent sonographic feature of severe aneuploidies, particularly Trisomy 18 and Trisomy 13 [25].

##### Association with Other Anomalies

Beyond aneuploidy, umbilical cord pseudocysts are also frequently linked to a range of structural congenital defects. These include abdominal wall defects, most notably omphalocele, as well as patent urachus, fetal hydrops, and various other anomalies [25]. The presence of a pseudocyst on a prenatal ultrasound should therefore be considered a significant “red flag”, prompting a thorough and detailed anatomical survey of the fetus.

##### Mucoid Degeneration

Mucoid degeneration is a histopathological term that describes a process of breakdown and liquefaction of the Wharton’s jelly matrix. It is not merely a descriptive term but represents a key pathogenic process underlying other, more grossly apparent abnormalities. The very first case of absent Wharton’s jelly reported in the literature in 1961 was described by the authors as “mucoid degeneration of Wharton’s jelly”, highlighting the historical and pathological link between these conditions [28]. This degenerative process is now understood to be the direct cause of pseudocyst formation, where the liquefied matrix accumulates to form a fluid-filled cavity [29].

This connection suggests the existence of a pathological continuum. The fundamental disease process appears to be a localized failure or enzymatic breakdown of the complex extracellular matrix of Wharton’s jelly. If this process results in the accumulation of fluid within a contained space, it manifests sonographically and pathologically as a pseudocyst. However, if the degenerative process is more aggressive, or if it is accompanied by a failure of the surrounding amniotic sheath to contain the liquefaction, it could progress to a complete focal loss of the jelly, resulting in a segment of exposed, unprotected umbilical vessels. This conceptual framework implies that the various structural pathologies of WJ—mucoid degeneration, pseudocyst formation, and segmental absence—may not be entirely distinct entities but rather different manifestations of a shared underlying process of matrix instability. Such a perspective has important clinical implications. The prenatal detection of a large or persistent pseudocyst should trigger consideration not only of the risk for aneuploidy but also of the possibility of a profound structural weakness in that segment of the umbilical cord. This could warrant heightened surveillance for signs of cord compression or compromised fetal circulation, as the cyst may signal a “forme fruste” of a more catastrophic structural failure.

The following table provides a clinicopathological summary of the major pathologies affecting Wharton’s jelly, synthesizing the key features, associated conditions, and potential outcomes to serve as a clinical reference [Table 1].

#### 2.4.2. Impact on Fetus During Pregnancy and Neonatal Outcomes

The pathological alterations in Wharton’s jelly have profound and direct consequences for the fetus and neonate. The nature and severity of the outcome are closely tied to the specific type and extent of the WJ pathology, ranging from catastrophic events like stillbirth to more chronic conditions such as fetal growth restriction and intrapartum complications.

#### 2.4.3. Catastrophic Outcomes: Stillbirth, Fetal Demise, and Perinatal Mortality

The most severe consequence of Wharton’s jelly pathology is fetal or perinatal death. The link is most stark and unequivocal in cases of segmental or complete absence of the jelly. Multiple case reports and pathological studies have documented this rare anomaly as a direct and demonstrable cause of stillbirth [7]. The pathophysiology is straightforward and mechanical: the loss of the protective WJ matrix leaves the umbilical vessels unprotected and susceptible to acute compression and occlusion [7,30]. This can occur during normal fetal movement, uterine contractions, or descent through the birth canal, leading to a sudden cessation of blood flow and acute fetal hypoxia and demise.

This understanding provides a crucial mechanistic basis for a subset of stillbirths that are often vaguely categorized as “cord accidents” [2]. The presence of a common cord entanglement, such as a nuchal cord or even a true knot, may not be inherently lethal. However, in the context of a structurally compromised umbilical cord—one with a focal area of absent or significantly reduced WJ—such an entanglement can become a fatal event. A systematic review and meta-analysis of 145 studies found that the presence of a true umbilical cord knot was associated with a significantly higher likelihood of stillbirth, with an odds ratio (OR) of 4.65 (95% CI 2.09–10.37) [31]. It is highly plausible that this risk is magnified when there is insufficient Wharton’s jelly to cushion the knot and prevent it from tightening to the point of complete vascular occlusion.

The prognosis associated with umbilical cord pseudocysts also encompasses these severe outcomes. While an isolated cyst found in the first trimester typically carries a good prognosis, the clinical course for fetuses with cysts that persist into the second and third trimesters is often dictated by the severe chromosomal or structural defects with which they are associated. In a retrospective review of 13 fetuses with umbilical cord pseudocysts, all seven of the chromosomally abnormal fetuses and two euploid fetuses with associated structural defects died either in utero or in the neonatal period [25]. This highlights that while the cyst itself may not be the direct cause of death, its presence serves as a powerful marker for underlying conditions that are often incompatible with life.

#### 2.4.4. Perinatal Morbidity and Compromised Development

##### Fetal Growth Restriction (FGR) and Placental Insufficiency

Beyond acute, fatal events, pathologies of Wharton’s jelly are significantly associated with chronic conditions that impair fetal development, most notably Fetal Growth Restriction (FGR). A study that measured the cross-sectional area of WJ using prenatal ultrasound found a statistically significant association between a decreased WJ area and low birth weight (*p* = 0.002) and the delivery of a small for gestational age (SGA) neonate [2]. This association is biologically plausible, as a thin umbilical cord with deficient WJ is more vulnerable to chronic, intermittent compression over the course of the pregnancy. Such repeated, sub-lethal insults to blood flow can impair the consistent delivery of oxygen and nutrients, leading to a state of chronic placental insufficiency and resulting in restricted fetal growth [15].

A particularly insightful finding from recent research is the strong positive correlation between the area of Wharton’s jelly and the overall dimensions of the placenta, including its width, length, and surface area. The same study also found that a decreased WJ area was significantly associated with the presence of clinically significant placental pathology (*p* = 0.01) [2]. This evidence suggests that the health and size of Wharton’s jelly may serve as a barometer for the health and functional capacity of the entire feto-placental unit. The placenta and umbilical cord develop in concert, and it is logical that their growth would be proportionally linked. A primary placental pathology, such as maternal vascular malperfusion, might create a suboptimal intrauterine environment that stunts the growth of both the fetus and its supporting structures, including the umbilical cord and its jelly. Conversely, a primary developmental defect in the umbilical cord, including inadequate WJ formation, could lead to abnormal hemodynamics that secondarily impair placental growth and function. Regardless of the initial insult, the amount of Wharton’s jelly appears to scale with the functional capacity of the placenta. This relationship implies that the sonographic measurement of WJ area, for which gestational age-specific nomograms have been developed, could potentially be refined into a novel, non-invasive clinical tool to assess placental function and predict the risk for FGR, thereby complementing existing methods like uterine and umbilical artery Doppler velocimetry [2,32].

##### Intrapartum Complications and Neonatal Admission

Abnormalities of Wharton’s jelly are also linked to a higher incidence of complications during labor and delivery. Cord constrictions resulting from WJ absence have been implicated in fetal intolerance to labor, as the unprotected vessels cannot withstand the compressive forces of uterine contractions [7]. This often manifests as abnormal or non-reassuring intrapartum fetal heart rate tracings, such as variable decelerations or profound bradycardia, which frequently necessitate emergency operative delivery [33].

A compelling case report detailed the intrapartum course of a pregnancy with a segmental absence of Wharton’s jelly. The patient presented with reduced fetal movements, and the admission cardiotocograph (CTG) rapidly deteriorated into a sustained, non-recovering fetal bradycardia, prompting a category 1 emergency cesarean section [33]. Although the infant was delivered alive, the period of acute hypoxia resulted in the passage of thick meconium, leading to meconium aspiration syndrome and requiring a 14-day admission to the neonatal intensive care unit (NICU) [33]. This case illustrates the acute nature of the complications and the potential for significant neonatal morbidity even when fetal demise is averted. More broadly, thin umbilical cords due to WJ deficiency have been associated with poor 5 min Apgar scores and an increased rate of NICU admission, reflecting the cumulative impact of chronic and acute circulatory compromise [15].

#### 2.4.5. Association with Congenital and Chromosomal Anomalies

There is a robust and well-documented association between certain structural pathologies of Wharton’s jelly and the presence of major congenital and chromosomal anomalies in the fetus. This link is most pronounced for umbilical cord pseudocysts. The clinical risk is highest when these cysts are multiple, when they persist beyond the first trimester into the second and third trimesters, or when they are found in conjunction with other abnormal sonographic markers [25].

The aneuploidies most frequently reported in association with umbilical cord pseudocysts are Trisomy 18 (Edwards syndrome) and Trisomy 13 (Patau syndrome), both of which are severe, life-limiting conditions [25]. The finding of a pseudocyst, particularly one with high-risk features, is therefore a strong indication for offering invasive prenatal diagnostic testing, such as amniocentesis, to determine the fetal karyotype [25].

In addition to chromosomal abnormalities, pseudocysts are also associated with a variety of structural defects. The most common of these are abdominal wall defects, particularly omphalocele, and anomalies of the urinary system, such as a patent urachus, where an embryological connection between the bladder and the umbilicus fails to close [25]. It is hypothesized that in cases of a patent urachus, fetal urine can track into the umbilical cord, causing localized swelling and liquefaction of the Wharton’s jelly, leading to pseudocyst formation [34]. Other associated anomalies include cardiac defects and fetal hydrops. The discovery of an umbilical cord cyst during a prenatal ultrasound mandates a comprehensive and meticulous anatomical survey of the entire fetus to search for these and other potential malformations.

#### 2.4.6. Diagnosis

The clinical approach to Wharton’s jelly pathologies involves prenatal diagnosis, primarily through ultrasonography, followed by risk stratification, appropriate fetal surveillance, and careful delivery planning. However, the efficacy of this approach is highly variable and depends on the specific pathology, with some conditions being readily detectable while others remain tragically elusive.

#### 2.4.7. Prenatal Diagnosis: The Role and Limitations of Ultrasonography

##### Sonographic Assessment

Two-dimensional ultrasonography is the cornerstone of prenatal evaluation of the umbilical cord. Standard obstetric ultrasound protocols typically include an assessment of the number of umbilical vessels, documentation of the placental and fetal cord insertion sites, and the identification of any gross abnormalities such as cysts or masses [35,36]. A more detailed examination, often prompted by suspicion or performed in a high-risk setting, can include a qualitative and quantitative assessment of the Wharton’s jelly itself [36,37].

##### Measuring Wharton’s Jelly Area

The cross-sectional area of Wharton’s jelly can be measured sonographically. This is achieved by obtaining a clear transverse view of a free loop of the cord, measuring the total cross-sectional area of the cord, and then subtracting the measured cross-sectional areas of the two arteries and the vein [2,35]. To aid in the interpretation of these measurements, several studies have published nomograms and reference curves that chart the normal trajectory of WJ area expansion across gestation. These data consistently show a linear increase in the WJ area during the first and second trimesters, which then begins to plateau at approximately 32 weeks’ gestation [2,35]. These nomograms allow for the objective classification of a cord as having a reduced or excessive amount of WJ relative to gestational age norms.

##### Diagnosing Cysts and Edema

Umbilical cord cysts are typically visualized on ultrasound as well-defined, anechoic (black) structures within the substance of the cord [38]. Color Doppler imaging is essential to confirm that these structures are avascular and distinct from vascular anomalies like aneurysms. Umbilical cord edema can be suspected in the presence of a diffusely thickened, hyperechoic cord. In some reported cases, the development of pseudocysts from a background of increasing cord edema has been followed serially with ultrasound examinations over time [39,40].

##### The Diagnostic Challenge of Absent Wharton’s Jelly

Despite the capabilities of modern ultrasound, there is a critical and dangerous gap in prenatal diagnostic ability when it comes to the most lethal of these pathologies: the segmental absence of Wharton’s jelly. The literature is replete with statements describing this condition as “almost impossible to diagnose during prenatal ultrasound evaluations” [41]. It is an exceptionally rare finding, and in the vast majority of reported cases, the diagnosis was made only after delivery, often in the setting of an unexplained stillbirth or an acute, catastrophic intrapartum event [7].

This diagnostic failure stems from several factors. Standard ultrasound screening protocols do not mandate, nor is it always feasible to perform, a meticulous, inch-by-inch survey of the entire length of the free-floating umbilical cord to assess the integrity of its surrounding jelly [36]. A short segment of absent WJ, perhaps only a centimeter in length, may not significantly alter the overall cord diameter or appearance and can be easily missed, particularly if the cord is coiled or if fetal positioning is suboptimal. This creates a scenario of a “silent” pathology. The structural defect is present, but it may not produce any chronic, detectable sonographic or Doppler abnormalities. The condition may only become clinically apparent when a specific mechanical trigger—such as fetal movement causing torsion at the weak point, or a uterine contraction compressing the unprotected vessels—occurs. This leads to an acute, rather than a chronic, event, manifesting as a sudden fetal bradycardia or demise. This limitation in current surveillance methods suggests that some cases of stillbirth classified as “unexplained” may, in fact, be due to this undetectable pathology. It raises important clinical questions about the management of pregnancies with persistent but non-specific warning signs, such as recurrent maternal perception of reduced fetal movements, even in the face of a reassuring CTG, as was the prelude to the acute event in one well-documented case report [33]. Although ultrasound remains the gold standard for evaluation, MRI is increasingly recognized as a valuable complementary tool—particularly in situations where ultrasound is limited or when a more detailed anatomical assessment is needed—potentially enhancing perinatal care [40,42].

### 2.5. Fetal Surveillance and Management

#### 2.5.1. Management of Cord Cysts

The prenatal detection of an umbilical cord cyst necessitates a structured management pathway to stratify risk and guide care.

1. **Detailed Anatomical Survey:** The first step is a comprehensive, high-resolution ultrasound examination to meticulously search for any associated fetal structural anomalies [25].
2. **Karyotyping:** Given the strong association with aneuploidy, invasive genetic testing via amniocentesis or chorionic villus sampling should be offered, particularly if the cysts are multiple, large, complex, persistent beyond the first trimester, or found in conjunction with any other sonographic marker of aneuploidy [43].
3. **Serial Surveillance:** For pregnancies that continue, especially those with large or numerous cysts, serial ultrasound surveillance is recommended. These follow-up scans, typically performed every four weeks, are used to monitor fetal growth for any signs of FGR and to assess the cysts for any change in size or evidence of umbilical vessel compression [34].
4. **Delivery Planning:** Advanced delivery planning is imperative. The potential for sudden vascular compromise and fetal demise means that decisions regarding the timing and mode of delivery must be carefully considered, often involving consultation with maternal-fetal medicine specialists. Continuous intrapartum electronic fetal monitoring is essential [34].

#### 2.5.2. Management of Decreased/Absent Wharton’s Jelly

A significant challenge in managing decreased or absent WJ is the rarity of a prenatal diagnosis. Consequently, there are no established, evidence-based management protocols. Clinical management is, therefore, often reactive rather than proactive. The case report of a live birth despite segmental WJ absence provides a crucial learning point: the mother had presented twice with reduced fetal movements prior to the final admission, where an acute fetal bradycardia prompted an emergency cesarean section [33]. This experience strongly suggests that maternal reporting of reduced fetal movements, even if intermittent, should be taken with the utmost seriousness, as it may be the only subtle indicator of an underlying, otherwise undetectable, life-threatening cord pathology. In such cases, a lower threshold for heightened surveillance or even delivery may be warranted.

#### 2.5.3. Management of Excessive Wharton’s Jelly/Edema

The literature does not provide specific guidelines for the clinical management of excessive Wharton’s jelly itself. The management approach is typically directed at the underlying associated condition, such as optimizing glycemic control in cases of maternal diabetes or managing the complications of fetal hydrops [15]. Given the documented adverse hemodynamic changes in edematous cords, pregnancies with this finding would warrant heightened fetal surveillance, including regular non-stress tests and biophysical profiles, to monitor for any signs of fetal compromise [23].

## 3. Conclusions and Future Directions

This comprehensive review of the literature consolidates the evidence demonstrating that the structural integrity of Wharton’s jelly is a critical, active determinant of fetal well-being, not merely a passive supportive stroma. The spectrum of its pathologies is broad, ranging from quantitative variations like absence or excess to focal structural lesions such as pseudocysts and mucoid degeneration. Each of these conditions carries a distinct profile of associated risks and potential outcomes. The complete or segmental absence of Wharton’s jelly, though rare, represents a direct and often lethal threat to the fetus through vascular compression. The presence of umbilical cord pseudocysts, particularly when persistent or multiple, serves as a crucial sonographic marker for severe underlying fetal chromosomal and structural anomalies. Furthermore, a quantitative reduction in Wharton’s jelly area is emerging as a potential surrogate indicator of broader placental insufficiency and a risk factor for fetal growth restriction.

Despite these critical connections, this review has identified several significant gaps in the current body of knowledge and clinical practice.

- **Etiology:** The fundamental molecular, genetic, and environmental causes of abnormal Wharton’s jelly development remain largely unknown. The pathways that lead to matrix degeneration, hypoplasia, or hyperplasia are poorly understood.
- **Prenatal Diagnosis:** A major deficiency exists in the ability of current ultrasound technology and screening protocols to reliably diagnose segmental absence of Wharton’s jelly prenatally. This “silent” pathology is responsible for some of the most catastrophic outcomes, yet it typically evades detection until after the adverse event has occurred.
- **Standardized Protocols:** There is a notable lack of consensus among international obstetric and ultrasound societies regarding the required depth and detail of a routine umbilical cord examination. Furthermore, for the majority of Wharton’s jelly pathologies, no standardized, evidence-based management protocols exist to guide clinicians in surveillance and delivery planning.

Addressing these gaps is essential for improving perinatal outcomes. Based on this review, the following recommendations for future research and clinical practice are proposed:

- **Improved Diagnostics:** Research efforts should be directed toward developing and validating novel diagnostic techniques that could improve the prenatal detection of subtle or segmental Wharton’s jelly abnormalities. This could include the application of high-frequency ultrasound transducers, advanced 3D/4D rendering techniques to better visualize the entire cord structure, or the investigation of potential biochemical markers in maternal serum or amniotic fluid that may reflect abnormal matrix turnover.
- **Prospective Studies:** There is a pressing need for large-scale, prospective, multicenter studies. Such studies are required to establish the true incidence of these pathologies, to identify risk factors, and to develop robust models for risk stratification. Specifically, studies that prospectively correlate sonographic measurements of Wharton’s jelly area with detailed postpartum placental pathology and long-term neonatal outcomes are needed to validate its potential use as a clinical marker of placental function.
- **Basic Science Research:** A renewed focus on the basic developmental biology of the umbilical cord is essential. Investigating the genetic and molecular pathways that govern the formation, maintenance, and degradation of the Wharton’s jelly matrix will provide the foundational knowledge needed to understand how and why these processes fail.
- **Guideline Harmonization and Development:** An international collaborative effort among professional societies is needed to harmonize clinical guidelines for the routine sonographic assessment of the umbilical cord. Consideration should be given to recommending a more detailed evaluation of Wharton’s jelly, particularly in pregnancies identified as high-risk for placental insufficiency or stillbirth. As more evidence becomes available, the development of standardized management algorithms will be crucial to ensure that pregnancies complicated by these pathologies receive optimal, evidence-based care.

## Figures and Tables

**Table 1:** The Spectrum of Wharton’s Jelly Pathologies: A Clinicopathological Summary.

Pathology	Key Sonographic and Histopathological Features	Associated Clinical Conditions (Maternal/Fetal)	Common Fetal/Neonatal Outcomes
**Absence/Reduction (Thin Cord)**	Cord diameter <10th percentile; decreased WJ area on ultrasound; constriction/stricture; exposed vessels; absent or attenuated matrix on histology [7].	Single umbilical artery (SUA), early-onset preeclampsia, gestational diabetes, oligohydramnios [2]	Stillbirth, perinatal death, fetal growth restriction (FGR), fetal intolerance to labor, non-reassuring fetal heart rate, emergency cesarean section, low Apgar scores, NICU admission [2,14]
**Edema/Excess (Thick Cord)**	Cord diameter >90th percentile (>2 cm); giant cord (>5 cm); less dense, swollen WJ matrix; potential for cavity formation and hemorrhages [15]	Maternal diabetes, hydrops fetalis, Beckwith-Wiedemann syndrome, chromosomal abnormalities (rare) [15]	Often benign if isolated; outcomes are typically related to the underlying condition. Severe edema can cause vascular compression leading to fetal distress or demise [23]
**Pseudocysts**	Anechoic, avascular, non-epithelial lined space within WJ; can be single or multiple, variable in size and location [27]	Trisomy 18, Trisomy 13, omphalocele, patent urachus, hydrops, other structural anomalies [25]	Favorable if isolated and transient in the first trimester. High risk of miscarriage, intrauterine death (IUD), and neonatal complications if persistent, multiple, or associated with other anomalies [25]
**Mucoid Degeneration**	Histopathological finding of matrix liquefaction and breakdown; precursor to pseudocysts and potentially segmental absence of WJ [28]	Considered the underlying process for pseudocysts and some cases of absent WJ.	Threatens fetal life by compromising the structural integrity of the cord, leading to the risks associated with pseudocysts and absent WJ [28]

## Data Availability

No new data were created or analyzed in this study. Data sharing is not applicable to this article.
